# Spontaneous resolution of acute gout: mechanisms and therapeutic targets

**DOI:** 10.1136/rmdopen-2023-003586

**Published:** 2023-09-21

**Authors:** Meiling Shi, Jiao Luo, Liting Ding, Lihua Duan

**Affiliations:** 1 Medical College of Nanchang University, Nanchang, China; 2 Department of Rheumatology and Clinical Immunology, Jiangxi Provincial People's Hospital, The First Affiliated Hospital of Nanchang Medical College, Nanchang, China; 3 Jiangxi Provincial Clinical Research Center for Rheumatic and Immunologic Diseases, Nanchang, People's Republic of China; 4 Key Laboratory of Rheumatology and Immunology, Health Commission of Jiangxi Province, Nanchang, People's Republic of China

**Keywords:** gout, tumor necrosis factors, interleukin 1 receptor antagonist protein, cytokines

## Abstract

Gout is a common inflammatory arthritis that has been increasing in both prevalence and incidence. Consequently, management of refractory and chronic gout has been gaining attention. Onset of gout is related to the deposition of monosodium urate crystals under hyperuricaemia. Interestingly, acute gout attacks often resolve spontaneously within 7–10 days, and many studies have confirmed the notion that gout flares can be self-relieved. However, the underlying mechanism for spontaneous remission of gout requires further elucidation. In this article, we summarise the roles and mechanisms related to spontaneous remission of gout, which are essential for understanding its pathogenesis and developing potential targeted therapies.

Gout caused by chronic deposition of monosodium urate (MSU) crystals is the most common inflammatory arthritis in adults. Gout flares are usually self-limiting inflammatory reactions that exhibit disappearance of redness, swelling and severe pain in the joints within 7–10 days, and may be associated with rapid induction of anti-inflammatory factors like Transforming growth factor beta 1 (TGFβ1), interleukin (IL)-10 and soluble tumour necrosis factor (TNF) receptors as well as intracellular cytokine negative regulators like cytokine inducible SH2-containing protein and regulatory immune cells.^1^ Nevertheless, the prevalence and incidence of refractory and chronic gout have continued to increase. Consequently, the question arises as to what can be learnt from the spontaneous remission of gout attacks that will aid the management of patients with refractory and chronic gout.

Research has shown that neutrophils are involved in not only the production of acute inflammation but also its self-remission. After phagocytosis of MSU crystals, neutrophils undergo the formation of neutrophil extracellular traps (NETs), a process known as NETosis, and release inflammatory cytokines. It is worth noting that neutrophils reach a threshold and that large DNA/MSU crystal structures, designated aggregation neutrophil extracellular traps (aggNETs), are subsequently formed. AggNETs sequester proinflammatory cytokines and chemokines for degradation by serine proteases, resulting in the relief of gout attacks.^2^ In 2021, CIT-013, which binds to citrullinated histones H2A and H4, was tested in a phase 1 clinical trial as a first-in-class humanised therapeutic antibody targeting NET formation and clearance. CIT-013 may be further developed for treatment of gout.^3^ Activated neutrophils that engulf dying neutrophils by phagocytosis in the late stages of gout attacks can promote the production of TGFβ1 and eliminate the inflammatory response.^4^ Furthermore, non-inflammatory phagocytosis of dying neutrophils by macrophages is associated with spontaneous remission of gout flares.^5^ Meanwhile, although studies of TGFβ1 in the treatment of autoimmune diseases have failed due to tumour development, single or a few doses may be beneficial for acute gout therapy.

IL-1β is an inflammatory cytokine secreted by macrophages that also plays a crucial role in gout. Canakinumab was found to be effective and well-tolerated for the short-term control of acute gouty arthritis in several clinical trials.^6^ Uptake of MSU crystals by macrophages induces activation of NOD-like receptor thermal protein domain associated protein 3 (NLRP3) inflammasomes, which in turn leads to activation of caspase-1 precursor (procaspase 1) that converts pro-IL-1β to biologically active IL-1β. In the clinic, colchicine relieves gout inflammation by inhibiting MSU crystal activation in the NLRP3 inflammasome, thereby blocking the release of IL-1β.^7^ Anakinra, a recombinant human IL-1Ra, is the first biological agent approved for treatment of rheumatoid arthritis. It acts by blocking the biological activity of IL-1 through competitive inhibition of the binding between IL-1 and interleukin-1 type I receptor (IL-1RI). Interestingly, synovial fluid (SF) from patients with acute gout contains significantly higher levels of IL-1Ra than SF from patients with osteoarthritis. Furthermore, IL-1Ra is positively correlated with serum uric acid and inflammatory markers, and two randomised controlled trials reported the use of anakinra to treat gout flares.^8 9^

Patients with gout have significantly elevated levels of TNF-α. Furthermore, the combination of TNF-α and MSU causes significant induction of precursor IL-1β expression in neutrophils, while MSU stimulation alone fails to induce precursor IL-1β expression and IL-1β secretion by neutrophils.^10^ In addition, MSU can indirectly promote pro-IL-1β production through local cell necroptosis, leading to release of damage-associated molecular patterns (DAMPs) that stimulate macrophage or dendritic cell production of TNF-α.^11^ During the development of gout inflammation, the levels of soluble TNF receptor I (sTNFRI) and II (sTNFRII) are significantly elevated in the SF. A case report indicated that the TNF-α antagonist etanercept combined with febuxostat showed efficacy for refractory gout. In addition to treatment of rheumatoid arthritis and ankylosing spondylitis, etanercept is currently in clinical use for acute refractory gout attacks.^12 13^

CD14 is a proximal point in the innate immune response to MSU crystal uptake, caspase-1 activation and IL-1β production in macrophages. Remarkably reduced MSU uptake is observed in CD14 knockout mice.^14^ Membrane-bound CD14 (mCD14) and soluble CD14 (sCD14) cooperate with Toll-like receptors (TLRs) to facilitate innate immune responses.^15^ We found that mCD14 expression on peripheral blood mononuclear cells is significantly reduced in patients with gout. Furthermore, serum sCD14 levels are significantly reduced and have a positive correlation with C-reactive protein levels. CD14 expression is also directly reduced after MSU stimulation of monocytes/macrophages from healthy volunteers. Therefore, reduced CD14 production in MSU-induced inflammation may contribute to spontaneous remission,^16^ and blockade of CD14 function may be useful for refractory gout. In fact, IC14, a chimeric monoclonal antibody, is a potential anti-CD14 therapeutic agent that has been investigated in COVID-19 patients.^17^ Thus, IC14 may also be useful for treatment of gout by blocking the interactions of CD14, DAMPs and MSU, thereby attenuating the NLRP3/IL-1β pathway.

The IL-1 family members IL-33 and IL-37 are known to be negative regulators of IL-1β signalling, and their anti-inflammatory properties have been demonstrated in numerous disease models. We previously showed that IL-33 is increased in patients with gout and that exogenous IL-33 reduces neutrophil recruitment and IL-1β production in MSU-induced acute inflammation,^18^ although IL-33 can attenuate sepsis by enhancing neutrophil influx to infection sites in experimental sepsis induced by cecal ligation and puncture.^19^ The IL-37 levels in patients with tophaceous gout are significantly increased, and patients who are carriers of distinct rare IL37 variants affecting the IL-37 protein structure and consequently its anti-inflammatory function experience either onset of gout at a younger age or a more severe disease phenotype. Moreover, administration of recombinant IL-37 can dampen the inflammation induced by MSU crystals.^20^ In addition to TGFβ1, these regulatory cytokines may be potential therapeutic agents for refractory gout.

## Conclusion

**Figure 1:**
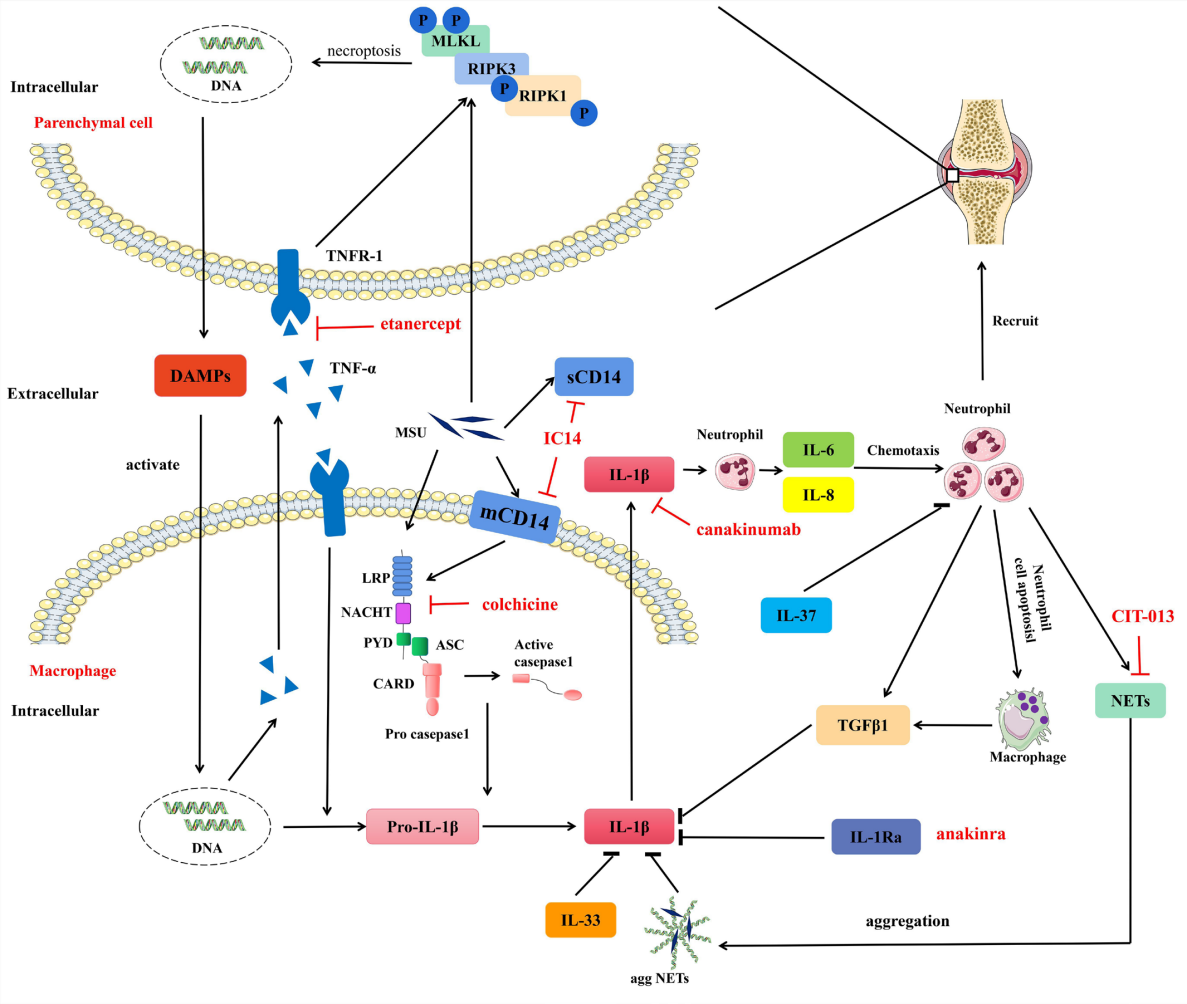
Summary diagram for the mechanisms of gout attacks and their self-remission. Phagocytosis of crystals by parenchymal cells activates immune cells surrounding parenchymal cells by inducing necroptosis leading to the release of DAMPs. Activated immune cells produce pro-inflammatory cytokines, such as pro-IL-1β and TNF-α. TNF-α can also induce necroptosis and promote the production of pro-IL-1β via TNFR1. The auto-amplification loop between cell death and inflammation can be blocked by using a soluble TNFR1-hIgG1 fusion protein etanercept. MSU crystals enter cells mainly through CD14, activate NLRP3 inflammasome and promote pro-IL-1β maturation. Elevated IL-33, IL-37, aggNETs, IL-1Ra, and TGFβ1 during inflammation relieve gout flares by inhibiting the action of the IL-1β pathway. These negative regulatory cytokines are potential targeted therapeutic drugs. In addition, anti-CD14 antibody (IC14) that blocks MSU uptake and colchicine that inhibits NLRP3 inflammasome activation, and anti-histone antibody (CIT-013) that inhibits NETs formation are also targeted therapies. aggNETs, aggregation neutrophil extracellular traps; DAMPs, damage-associated molecular patterns; IL, interleukin; mCD14, membrane-bound CD14; MSU, monosodium urate; NETs, neutrophil extracellular traps; NLRP3, NOD-like receptor thermal protein domain associated protein 3; TGFβ1,Transforming growth factor beta 1; TNF, tumour necrosis factor; TNFR-I, TNF receptor I.

At present, the number of patients with refractory gout is continuing to increase, and the question of how to control acute attacks in patients with refractory gout is a clinical problem that requires urgent resolution. Treatment of acute gout inflammation mainly relies on non-steroidal anti-inflammatory drugs, colchicine and glucocorticoids; however, refractory gout is a persistence of clinical manifestations characterised by the inability to reduce the serum urate acid concentration below the target 6.0 mg/dL, increased MSU deposition and tophi formation, and chronic inflammatory arthritis, ongoing symptoms of recurrent flares. Patients with refractory gout often do not respond well to these drugs or have contraindications. Targeted therapy, including biologics and targeted synthetic small molecule drugs, has been widely used in other inflammatory arthritis diseases, such as rheumatoid arthritis and ankylosing spondylitis, with promising results. We hope that patients with refractory gout can receive similar targeted therapies to patients with rheumatoid arthritis and ankylosing spondylitis. We have discovered many potential therapeutic targets by elucidating the pathogenesis and self-remission mechanisms of gout (figure 1). Fortunately, some drugs have either received approval or are currently in clinical trials. We believe that targeted therapy for gout is coming soon, providing welcome relief for patients with refractory gout.
